# Differentiation of Mouse Primordial Germ Cells into Functional Oocytes *In Vitro*

**DOI:** 10.1007/s10439-017-1815-7

**Published:** 2017-02-27

**Authors:** Kanako Morohaku, Yuji Hirao, Yayoi Obata

**Affiliations:** 1 Department of Bioscience, Tokyo University of Agriculture, 1-1-1 Sakuragaoka, Setagaya-ku, Tokyo, 156-8502 Japan; 2 Institute of Livestock and Grassland Science, NARO, 2 Ikenodai, Tsukuba, Ibaraki 305-0901 Japan

**Keywords:** Primordial germ cell, *In vitro* oocyte growth, Oocyte preservation

## Abstract

Various complex molecular events in oogenesis cannot be observed *in vivo*. As a bioengineering technique for female reproductive tissues, *in vitro* culture systems for female germ cells have been used to analyze oogenesis and preserve germ cells for over 20 years. Recently, we have established a new methodological approach for the culture of primordial germ cells (PGCs) and successfully obtained offspring. Our PGC culture system will be useful to clarify unresolved mechanisms of fertility and sterility from the beginning of mammalian oogenesis, before meiosis. This review summarizes the history of culture methods for mammalian germ cells, our current *in vitro* system, and future prospects for the culture of germ cells.

## Introduction

In mice, primordial germ cells (PGCs) first emerge at around 7.5 days post-coitum (dpc).^27^ They are defined by high levels of tissue-nonspecific alkaline phosphatase activity and/or as *Dppa3/PGC7/stella*-positive cells at the base of the allantois.^83^ PGCs are specified by *Blimp1/Prdm1* and *Prdm14* expression prior to 7.5 dpc.^69^,^104^ They migrate into gonads with the help of chemotaxis factors, such as c-kit/Kit and kit ligand/Kitl,^15^,^26^,^110^,^111^ until 10.5 dpc and rapidly proliferate from approximately 40 to 25,000 in number between 7.5 and 13.5 dpc.^95^ During this period, PGCs become progressively different from their ancestors; over time, they exhibit the repression of genes characteristic of their neighboring somatic cells,^83^ reprogramming including the erasure of genomic imprinting,^41^,^61^,^88^,^89^ and the acquisition of sexuality.^1^,^36^,^57^,^84^ Consequently, they are ready for oogenesis or spermatogenesis.

Oogenesis begins with the differentiation of isomorphic PGCs into oogonia following sexual differentiation. Mammalian PGCs and oogonia mitotically divide and reach a maximum number at the fetal stage (Table 1).^5^,^9^,^11^,^29^,^34^,^48^,^49^,^54^,^58^,^64^,^79^,^106^ In female mouse embryos, PGCs receive retinoic acid signals from the adjacent mesonephros and *Stra8* expression is then induced.^44^,^73^ STRA8 requires pre-meiotic DNA replication.^6^ As a result, PGCs cease proliferation and enter meiosis at around 14.5 dpc in females, but are arrested at G1/G0 in the mitotic stage until a few days after birth in males.^55^ It has been thought that all oogonia are destined to enter meiosis in fetal ovaries, after which more than half of oocytes are lost by apoptosis.^5^,^9^,^64^ Surviving oocytes are assembled into primordial follicles. These primordial follicles become dormant and only a small proportion are activated to produce fully matured oocytes at the adult stage. The limited number of mature oocytes represents a disadvantage for breeding, reproduction, and scientific research. Furthermore, the regulatory mechanisms of mammalian oogenesis remain largely unknown.

**Table 1:** The numbers of germ cells in mammalian species.

Species	Estimated maximum total no. of germ cells in the ovary		Estimated total no. of germ cells in the ovary at birth	Estimated total no. of germ cells in the ovary at puberty	References
	Approx. no. of germ cells	Stage			
Mouse (C57BL/6)	15,000	15.5 dpc	7000	3000–5000	11, 64
Rat	75,000	18.5 dpc	52,000	5000–10000	9, 49
Human	5 × 10^6^–7 × 10^6^	Midgestation	5 × 10^5^–1 × 10^6^	1.5 × 10^5^–3 × 10^5^	5, 29, 48
Rhesus monkey	–	–	4 × 10^5^	–	34
Bovine	2 × 10^6^	Prenatal	1.2 × 10^5^–1.5 × 10^5^	–	106
Sheep	9 × 10^5^	Day 75 of gestation	–	30,000–50,000	79
Pig	8 × 10^5^–1.2 × 10^6^	Day 90 of gestation	4.5 × 10^5^	–	58

*In vitro* systems have helped elucidate mechanisms underlying PGC specification, proliferation, and differentiation. Recently, we successfully demonstrated the complete *in vitro* generation of fertile mouse oocytes from PGCs for the first time.^63^ Such an *in vitro* system is expected to unravel the mechanisms of oogenesis and preserve female gametes. In this review, we describe the brief history of the *in vitro* systems for recapitulating germ cell development and summarize the development and current state of these cutting-edge techniques for PGC/oocyte culture. We also discuss potential future applications of our advanced technique, e.g., for large-scale oocyte production, identification of the requirements for fertile oocytes, and visualization of oogenesis.

## History of PGC Culture in Mice

Early studies on germ cell culture focused on determining how mammalian PGCs migrate into gonads and subsequently differentiate into oocytes. In the 1980s, Tam and Snow removed small pieces of the primitive streak containing the future PGCs-fated region at 6.5 and 7.5 dpc and cultured them in DMEM on plastic dishes owing to the difficulties in tracing PGC fate *in vivo*. The small pieces increased in size after 24 h of culture, but growth was arrested at 48 h.^95^ McLaren and colleagues isolated PGCs from female gonads at 13.5 dpc, and tried to culture them *in vitro* without feeder cells. These PGCs survived and progressed into meiosis, suggesting that female PGCs at 13.5 dpc are committed to enter meiosis, independent of the gonadal environment.^17^ Later, it was found that the culture of isolated PGCs on STO cells (a mouse embryonic fibroblastic cell line) effectively extends PGC survival and enables the successful recapitulation of PGC migration *in vitro*.^19^,^93^ STO cells produce various key factors for PGC proliferation, such as kit ligand (also known as stem cell factor (SCF) or steel factor) and leukemia inhibitory factor (LIF), at around 8.5–11.5 dpc.^52^ The importance of STO cells for PGC culture can be explained by the phenotypes and genotypes in *W/W* and *Sl/Sl* mutant mice, which are sterile because PGCs are incapable of migration into gonads and proliferation. It was found that the *W* locus encodes c-kit/*Kit*, a receptor for the kit ligand, in 1988 and the *Sl* locus encodes kit ligands/*Kitl*, in 1990.^15^,^26^,^110^,^111^

Recent studies have concentrated on PGC specification. Yoshimizu *et al.* cultured epiblasts from 5.5-dpc embryos with or without extra-embryonic tissues, demonstrating that PGC emergence requires extra-embryonic tissues.^107^ PGC generation from proximal epiblasts requires BMP4 from extra-embryonic tissues.^45^ Breakthrough experiments performed by Saitou and colleagues have shown that PGC-like cells (PGCLCs) are successfully differentiated *in vitro* from epiblasts of 6.0-dpc embryos in which PGCs are not specified.^68^ They found that BMP4 and WNT3 are indispensable for the activation of *Blimp1* and *Prdm14* in the posterior part of the proximal epiblast from which PGCs arise. WNT3 induces T(BRACHYURY) expression, leading to the activation of *Blimp1* and *Prdm14*. Both *Blimp1* and *Prdm14* are transcriptional repressors essential for the loss of somatic cell fate and PGC specification.^3^ After PGC specification, BMP4, BMP8b, LIF, Kit ligand, and EGF enhance the proliferation of PGCLCs *in vitro*. PGCLCs exhibit the erasure of genomic imprinting. Consequently, they develop into functional sperm following transplantation to beneath the tunica albuginea of adult testes.^68^ Interestingly, PGCs proliferate *in vitro*, but their growth is arrested at corresponding time points *in vivo.* 19,28,52,68

In the presence of basic fibroblast growth factor (bFGF), Kit ligand, and LIF, PGCs are reprogrammed and acquire pluripotency and infinite proliferation activity.^53^,^78^,^81^ These cells are called embryonic germ (EG) cells and are no longer equal to PGCs. Recently, EG cells have also been established *via* the activation of serine/threonine kinase AKT,^51^ trichostatin A, histone deacetylase inhibitor,^21^ or inhibitors of mitogen-activated protein kinase signaling and of glycogen synthase kinase 3 (2i).^47^ *In vitro* systems to extend PGC proliferation while maintaining their intrinsic properties, have not been developed to date.

Culture methods for fetal gonads containing PGCs have also been established to examine the mechanisms of gonadal somatic cell and PGC differentiation. Until PGCs cease proliferation *in vivo*, gonadal somatic cells commit to the sexual differentiation of PGCs. Studies on the role of gonadal somatic cells in sexual differentiation have shown that the timing of meiotic progression in the indifferent gonads from 11.5-dpc embryos is altered by culture with ovaries or testes from 14.5-dpc embryos on 1% agar on a Nuclepore filter.^12^,^94^ Later, using a gas-liquid interface culture system in which gonads were cultured on a small block of 2% agar or on a micropore membrane filter with a thin layer of culture medium, more precise results were obtained. The culture of sexually chimeric gonads produced by the aggregation of XY gonadal somatic cells and XX germ cells or the opposite combination showed that the sex of germ cells is committed by gonadal somatic cells at 11.5–12.5 dpc in males and 12.5–13.5 dpc in females.^1^ This method also improved germ cell development, e.g., PGCs in the gonads obtained from 11.5-dpc female embryos were able to differentiate into oocytes with diameters of greater than 60 *μ*m after 23 days of culture.^56^ However, organ culture systems have not been designed to enable the completion of oogenesis or spermatogenesis. *In vitro* gametogenesis does not exactly recapitulate events that occur during gametogenesis *in vivo* unless fertile gametes are produced. Eppig *et al.* successfully cultured newborn ovaries containing non-growing oocytes and the derivative secondary follicles to obtain mature oocytes, which were able to develop to term after *in vitro* fertilization.^22^ Accordingly, they established a system with the potential to precisely recapitulate oogenesis. Ogawa *et al.* also demonstrated the cultivation of neonatal testes containing prospermatogonia on agar, yielding fertile sperm after intracytoplasmic sperm injection.^87^ However, the entire process of either oogenesis or spermatogenesis from PGCs to mature gametes has not been replicated *in vitro* in the 20 years since these studies.

## Completion of Mouse Oogenesis *In Vitro*

The production of fertile oocytes from PGCs, oogonia, or immature oocytes provides a basis for understanding the mechanisms of oogenesis in coordination with folliculogenesis and for preserving female gametes. Ovarian somatic cells are essential for the *in vitro* recapitulation of oogenesis.^39^ Ovaries consist of granulosa cells, theca cells, oocytes, and stromal cells. They produce numerous cytokines and steroid hormones to support oogenesis and self-organization *via* paracrine and autocrine signaling.^72^ These factors have not been comprehensively identified; accordingly, the culture of ovaries and/or follicles has been adopted for establishing an *in vitro* system, rather than the culture of oocytes alone, without their surrounded follicle cells, in livestock, rodents, nonhuman primates and humans.^4^,^24^,^39^,^92^,^96^

Generally, the developmental ability of oocytes grown *in vitro* is limited by long culture times and a lack of appropriate culture conditions. To overcome these difficulties, ovarian pieces derived from fetuses or juveniles are transplanted into adult mice, resulting in the successful production of larger quantities of high-quality oocytes from explanted grafts than are obtained *in vitro*.^91^ Several studies have shown that the xenogenetic transplantation of ovaries into immunodeficient mice induces oocyte growth.^7^,^10^,^60^,^71^ Thus, an* ex vivo* strategy may be beneficial when useful fetuses/animals die prior to birth/puberty or for the recovery of fertility in ovariectomized cancer patients. Yet, an * ex vivo* strategy cannot be used to produce functional oocytes as effectively as intact ovaries,^50^ cannot completely prevent the reintroduction of cancer cells in patients, and is less appropriate for studies of oogenesis because it is blinded to sequential changes in oogenesis.

Ovarian/follicular culture has been examined extensively in several mammals. Meiotically mature oocytes are successfully developed by the culture of preantral follicles, oocyte-granulosa complexes, or ovarian pieces in human, bovine, sheep, and pig.^8^,^14^,^38^,^65^,^70^,^103^ However, *in vitro* systems have poor outcomes depending on the length of the culture period required for the completion of oogenesis. Among mammals, mouse oocytes with proven fertility have been successfully produced from early-stage oocytes at comparably high efficiency *in vitro*.^31^,^59^,^62^,^66^ Live mouse pups have been obtained from the culture of secondary follicles derived from ovaries of juveniles and from a 2-step culture of neonatal primordial follicles, i.e., ovarian culture followed by follicle culture.^22^,^23^,^31^,^62^,^66^

Compared to the culture of immature oocytes embedded in the primordial or secondary follicles, a greater number of events in oogenesis need to be achieved in PGC culture.^4^,^24^,^92^,^96^ For example, prior to switching from mitosis to meiosis, female germ cells form cysts *via* incomplete cytokinesis. Oocytes cysts are broken after oocytes enter meiosis, and each oocyte is enclosed by a few flattened granulosa cells to form a primordial follicle; the first meiosis is then arrested at the diplotene stage of prophase I. Many studies have attributed female sterility to abnormalities in meiosis or follicle formation.^85^ A complete *in vitro* system for recapitulating oogenesis endows oocytes with totipotency and fertility. However, existing methods are not sufficient to reproduce oogenesis. The resultant oocytes do not reach the second meiosis or do not acquire ooplasmic competency to support full-term development.^18^,^67^,^90^,^109^

One issue is the long duration required for organ culture to produce fertile oocytes, i.e., 4 weeks or more. The ovaries/gonads are separated from the vasculature to supply nutrition and hormones from the mother, placenta, and neighboring/distal organs *via* endocrine systems and to support gas exchange.^17^,^56^,^109^ This causes low metabolic activity, degradation of supporting cells, and low-quality oocytes. Hence, the culture system needs to be switched to follicle culture from organ culture after each oocyte is enclosed by follicular cells. However, applying a 2-step culture system established for neonatal ovaries to fetal gonads containing PGCs has not been achieved. Conventional culture conditions cause hypoplasia of follicles in the gonads. Consequently, follicles cannot be isolated from cultured ovaries when the starting point for organ culture is prior to follicle formation *in vivo*.^67^,^91^,^109^ Even though many oocytes grow in size without being enclosed by a follicle structure, they never reach the functionally mature stage. This is a major limitation in the production of fertile oocytes from PGCs *in vitro*. We previously showed that nuclear transfer between *in vivo*-derived fully grown oocytes and *in vitro*-derived immature oocytes is needed to overcome the incompetence of oocytes differentiated from PGCs *in vitro*. Some reconstituted oocytes develop to offspring.^67^ However, a true *in vitro* system is required because nuclear transfer experiments mask the essential factors for the acquisition of oocyte competence and the mechanisms by which oocytes acquire competence, similar to * ex vivo* strategies.

**Figure 1:**
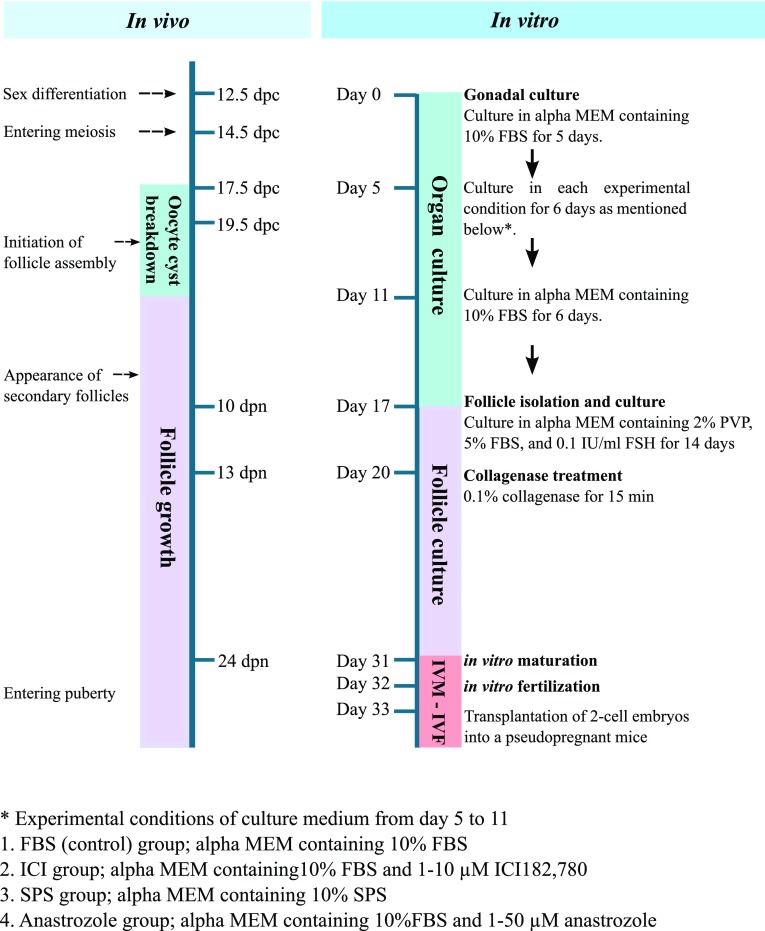
Timeline for PGC culture. Our culture system for PGCs is consisted of a gonadal culture and a follicle culture, and takes a month to obtain matured oocytes from 12.5-dpc embryonic gonads. We examined several culture conditions from day 5 to day 11: Gonads from embryos at 12.5-dpc embryos were cultured in 10% FBS-containing alpha MEM (FBS group), cultured in 10% FBS- and 1–10 *μ*M ICI-containing alpha MEM (ICI group), cultured in 10% SPS-containing alpha MEM instead of FBS (SPS group), and cultured in 10% FBS- and 1–50 *μ*M anastrozole-containing alpha MEM (anastrozole group). ICI group was the best culture condition to produce secondary follicles *in vitro*. Secondary follicles appeared in ovaries *in vitro* by day 17 of culture and were then isolated from ovaries mechanically for the follicle culture. At day 20, the follicles were treated with 0.1% collagenase, thereafter, they were cultured for another 9–11 days.

**Figure 2:**
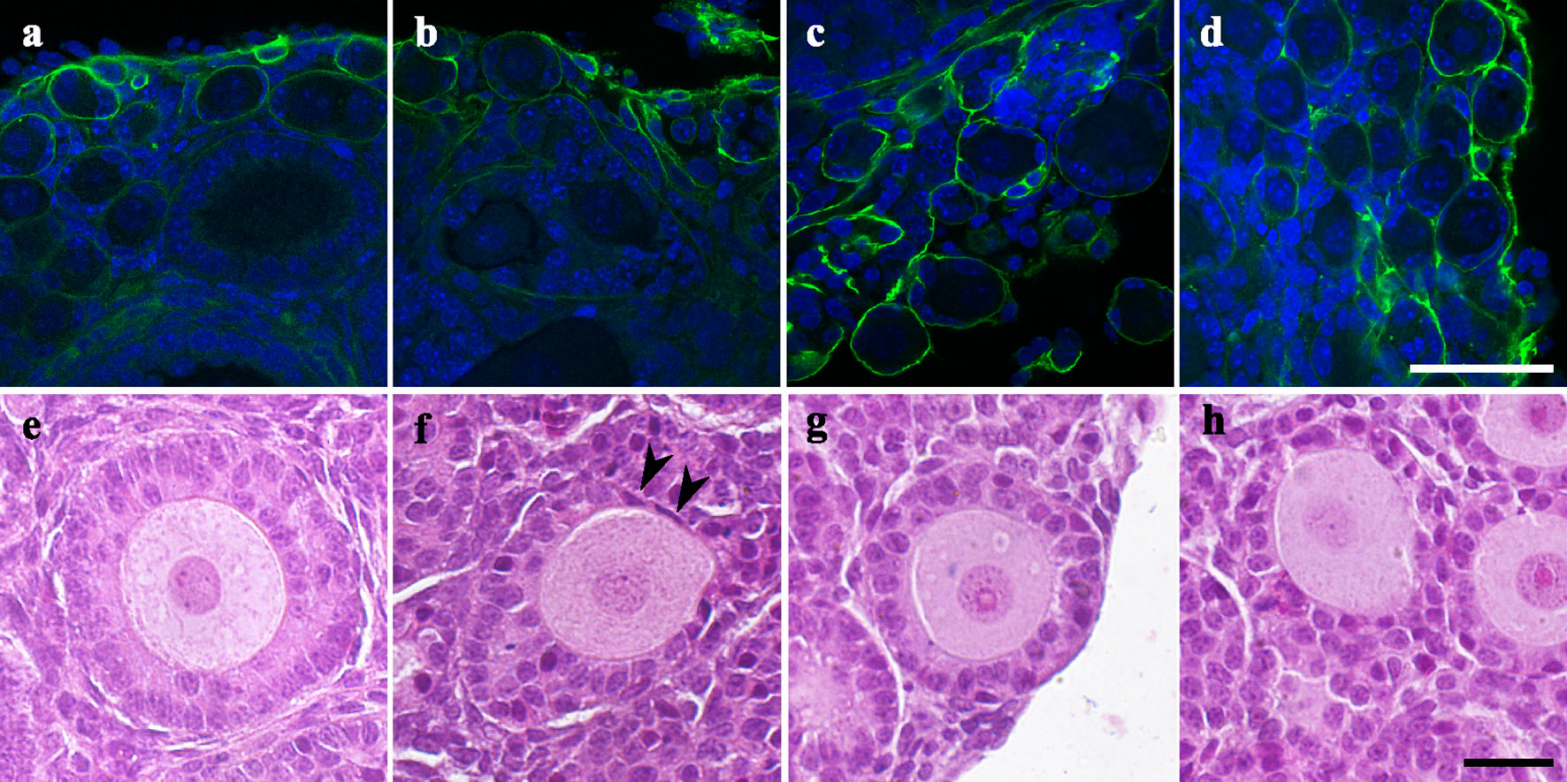
Morphology of *in vitro* grown follicles. (a–d) Immunofluorescence staining of the extracellular matrix. Ovaries derived from 10-dpn mouse (a), FBS group (b), ICI group (c), and SPS group (d). Each follicle was enclosed by laminin in both ICI and SPS groups but not in FBS group. Green, laminin; Blue, nuclei. (e–g) Histology sections of ovaries. Secondary follicles in the ovary of 10-dpn mouse (e), FBS group (f), and ICI group (g, h). Alignment of follicular cells was less regular in the ovaries of FBS group. Flattened theca like-cells attached to oocyte (black arrowheads) in FBS group (f). ICI group, secondary follicles were clearly formed (g), but the borders of some follicles were not clear (h). Bar = 50 *μ*m.

A breakthrough in PGC culture has come from the findings of Pepling and our studies.^16^,^63^ We adapted an ordinary 2-step culture system, which was established by Eppig and colleagues, to grow oocytes in newborn mouse ovaries for PGC culture^22^,^66^ (Fig. 1). Mouse embryonic gonads from 12.5-dpc embryos were cultured for 17 days on a Transwell-COL membrane within a thin layer of culture medium containing 10% fetal bovine serum (FBS). The number of isolated secondary follicles per ovary on day 17 of the culture was low (average, 6.2 follicles per ovary),^63^ consistent with previous results.^67^,^90^,^109^ A histological analysis of cultured ovaries showed multiple-oocyte follicles and the failure of each oocyte to be enclosed in the follicle. These results indicated abnormal follicle formation and explained the low yield of secondary follicles from embryonic ovaries (Fig. 2). To improve the failure of follicle development in the culture, we focused on oocyte cyst breakdown that occurs in the middle of the culture period. We surmised that the cytokinesis of oocytes is not completed or is delayed *in vitro*. Oocyte cyst breakdown occurs at or just prior to the time when a single oocyte is surrounded by granulosa cells.^76^ In previous reports by Pepling and colleagues, the introduction of estrogen into the organ culture medium prevented oocyte cyst breakdown in newborn mouse ovaries.^16^ Some reports have suggested an association between follicle formation in fetal ovaries and a decrease in estrogen *in vivo*.^105^ However, maternal- or placenta-derived estrogen is completely isolated by transferring fetal gonads to the *in vitro* environment. Therefore, to understand the molecular basis for abnormalities in follicle formation *in vitro*, we conducted RNA sequencing (RNA-seq) in fetal-derived ovaries after 7 days of culture and compared transcripts with those of neonatal ovaries on the corresponding day. An RNA-seq analysis showed that more than 500 genes are differentially expressed in *in vitro*-derived ovaries compared with neonatal ovaries. Interestingly, the most common upstream regulator of these differentially expressed genes was estrogen. Estrogen binds to estrogen receptor 1 (ESR1), estrogen receptor 2 (ESR2), and G protein-coupled estrogen 1 (GPER1). ESR1 and ESR2 in the presence of bound estrogen bind to estrogen response elements (5′-AGGTCAnnnTGACCT-3′) and regulate transcription.^43^ There was no evidence of substantial amounts of estrogen in the medium or that ESR1 and ESR2 were elevated in the *in vitro*-derived ovaries. Therefore, we hypothesized that 1) FBS contains estrogen-like factor(s) that can bind to ESR1 and/or ESR2, or 2) FBS contains many materials (e.g., cholesterol) needed to synthesize estrogen within the ovaries. To test these hypotheses, gonads from embryos at 12.5 dpc were cultured in medium supplemented with FBS for 17 days. From day 5 to day 11 when oocyte cyst breakdown occurs, an antagonist of both ESR1 and ESR2, ICI 182,780 (ICI), was added, an inhibitor of the aromatase, anastrozole, was added (unpublished data), or serum protein substitute (SPS) was added instead of FBS (Fig. 1). ICI 182,780 is known as fulvestrant and is used for breast cancer therapy to minimize estrogen activity.^82^,^97^ Anastrozole is also used for breast cancer therapy to inhibit estrogen production. The number of isolated secondary follicles was dramatically higher in the ICI-treated group and moderately higher in the SPS group compared to that in the FBS group (average, 82.0 follicles per ovary for 10 *μ*M ICI, 27.2 follicles per ovary for SPS, and 6.2 follicles in the FBS group). Immunohistochemical analysis showed that each oocyte was enclosed within the follicle with two or more layers of granulosa cells in the ICI and SPS groups (Fig. 2). Since anastrozole had no effect on the number of isolated secondary follicles (average, 2.3 follicles per ovary, unpublished data) and their phenotypes, ovaries would not produce excessive estrogen *in vitro*. Furthermore, the addition of estradiol to the medium containing ICI or SPS complementarily decreased the number of secondary follicles per ovary. Therefore, we concluded that the upregulation of estrogen signaling resulted in abnormal secondary follicle development *in vitro* and ICI addition to the medium for gonadal culture overcame this abnormality.

We also modified the follicle culture protocol established by Eppig in 1989.^23^ Our previous study showed that polyvinylpyrrolidone (PVP), a high-molecular-mass compound, improved follicle growth and survival *in vitro* in both bovines and mice.^37^,^62^ Therefore, we added 2% PVP to the medium for the follicle culture (Fig. 1). We observed a more striking impact of PVP on the follicles isolated from *in vitro*-derived ovaries than on those from *in vivo*-derived ovaries. The survival rate increased by at least 3 times by the addition of PVP to the medium. It is not clear why PVP is effective for increasing the survivability and growth of follicles. However, dextran, a representative macromolecular substance, has been used for organ preservation. We speculate that PVP might play a role in sustaining the structure of oocyte-surrounding follicle cells, maintaining their viability and preventing the diffusion of cytokines into the medium. In fact, PVP increased the mRNA expression of genes encoding cytokines, such as BMP6, BMP15, c-kit, and kit ligand, in follicles during culture.^63^

Another key to producing fertile oocytes from PGCs *in vitro* is collagenase treatment (Fig. 1). When we isolate secondary follicles from juvenile mice, ovaries are generally treated with collagenase.^23^ However, relatively fewer follicles were isolated after the collagenase treatment of *in vitro*-derived ovaries. This is attributed to the fragility of follicles from *in vitro*-derived ovaries and the random cellular alignment of some (Fig. 2f). We mechanically isolated secondary follicles, using a fine tungsten needle, and cultured intact follicles in medium containing 2% PVP. Follicles were able to grow; however, at the end of follicle culture, the layer of cumulus cells surrounding the oocyte was thin. The resultant oocyte could not develop beyond the 2-cell stage after fertilization.^63^ In contrast, collagenase treatment of mechanically isolated follicles exposed oocyte-granulosa cell complexes to the medium, resulting in an appropriate thickness of the cumulus cell layer surrounding the oocyte after follicle culture. The exposure of oocyte-granulosa cell complexes to the medium might promote gas exchange, nutritional intake, and the clearing of waste products *via* granulosa cells. The oocytes differentiated from PGCs *in vitro* grew to full size (approximately 80 *μ*m in diameter). Oocytes produced by this method exhibit successful fertilization, the completion of meiosis, and the acquisition of totipotency. Following the transplantation of 2-cell embryos in the oviducts of pseudopregnant mice, two to three pups per cultured gonad were born using our culture system. Pups from *in vitro* differentiated oocytes exhibited normal phenotypes and fertility. Oocyte-derived imprinting also persisted in these pups.^63^ Thus, a culture system for recapitulating oogenesis has been developed in a step-by-step manner.

## Widely Applicable Strategy to Produce Fertile Oocytes from PGCs

**Figure 3:**
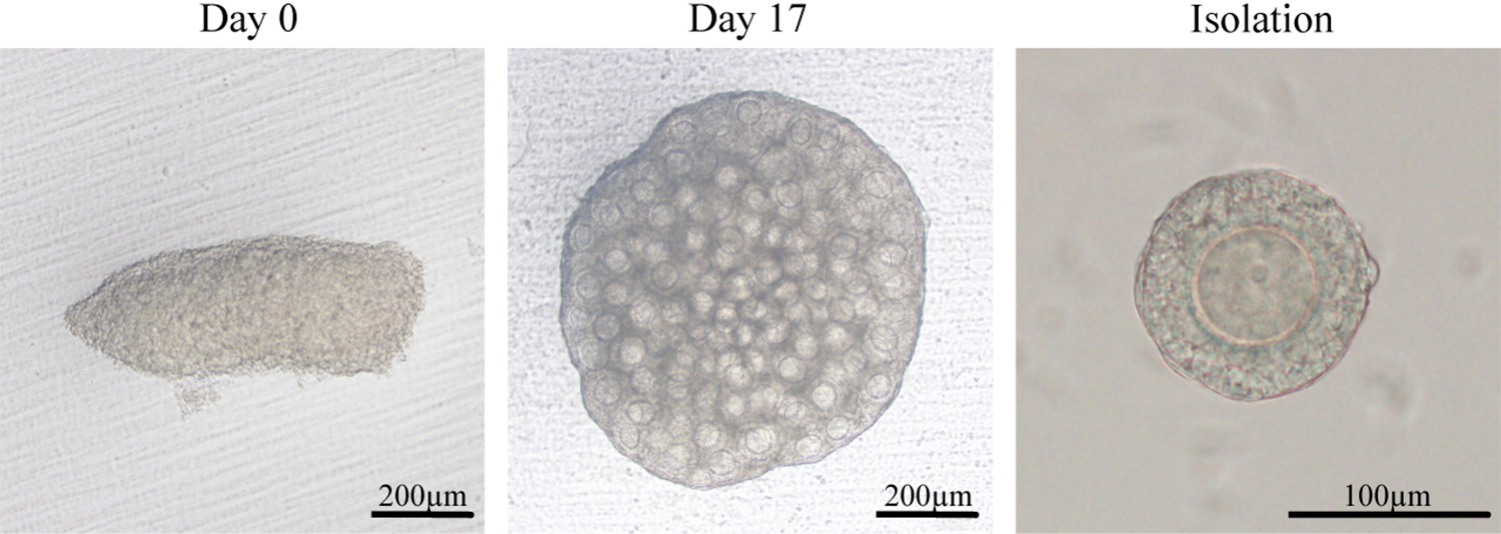
Vitrified-warmed gonads for production of oocytes. The gonad was cut into two or three pieces, dipped in the vitrification solution and frozen in liquid nitrogen (LN_2_). Bright-field images show thawed gonads cultured for 0 and 17 days. Bar = 200 *μ*m. A representative isolated follicle is labelled “isolation.” Bar = 100 *μ*m.

Vitrification is a useful technique for germ cell preservation. It is a kind of cryopreservation that avoids ice crystal formation by passing the cryohydric point quickly and therefore minimizes less cell damage.^80^ Vitrification as an alternative method for cryopreservation has been used for the preservation of oocytes, zygotes, and ovarian tissues in mice, bovines, humans, and so on.^2^ In our previous reports, functional oocytes and pups were successfully obtained from gonads vitrified/warmed following the method reported by Wang *et al*.^99^ In brief, ovaries derived from 12.5-dpc embryos were equilibrated for 20 min in vitrification medium containing 10% ethylene glycol, 10% dimethylsulfoxide (DMSO), and 4 mg/ml bovine serum albumin (BSA) in L15 base medium, and then for three minutes in 17% ethylene glycol, 17% DMSO, 0.75 M sucrose, and 4 mg/ml BSA. After equilibration, the gonads were transferred to a cryotube and vitrified at −196 °C in liquid nitrogen. A warming procedure was carried out in 0.5 M sucrose for 3 min at 37 °C, and for 2 min at room temperature. Then, the gonads were washed in 0.25 M sucrose, 0.125 M sucrose, and culture medium, in sequence. Following warming, the gonads were cultured using our above-described methods with ICI incorporated in the organ culture medium, PVP in the follicle culture medium, and collagenase treatment (Fig. 3). Although the efficiency by which secondary follicles were obtained was low compared with that for non-vitrified gonads, we successfully obtained pups from the culture of vitrified/warmed ovaries.

## Future Perspectives for PGC Culture

Many key factors determining the growth of oocytes and follicles have been identified, including kit ligand, GDF9, BMP4, BMP7, activin, inhibin, EGF, FSH, and so on.^62^ Granulosa and theca cells support oocyte growth by secreting factors, and oocytes also produce factors for follicle cell differentiation and proliferation. In our system, the medium contains FBS during the whole culture period prior to fertilization. We used alpha-MEM supplemented with ascorbic acid and ICI for gonadal culture, and ascorbic acid, PVP, and FSH for follicle culture. When 10% SPS, which consists of serum albumin and alpha, beta, and gamma globulins, was used for gonadal culture instead of 10% FBS throughout organ culture, gonad growth was restricted and only low-quality follicles with a thin layer of granulosa cells formed. Knockout serum replacement had the same effect or was inferior to SPS supplementation. The duration of the development of fertile oocytes from PGCs is very long, but oocytes do not undergo renewal. Therefore, the accumulation of tiny defects leads to a loss of fertility in oocytes. To establish an *in vitro* system for recapitulating oogenesis, for the first time, FBS cannot be excluded from the medium.

Eppig *et al.* established a chemically defined medium for follicle culture. It contains BSA, insulin, transferrin, selenium, FSH, EGF, and fetuin,^22^ and has been adopted for human follicle culture, with some modifications.^103^ In 1996, for the first time, the successful growth of oocytes capable of developing to offspring from neonatal ovaries was demonstrated following a 2-step culture, i.e., ovarian culture with FBS-containing medium for 8 days and follicle culture with chemically defined medium for 14 days, as described above. Although this was a substantial achievement, the culture of gonads or ovaries still requires FBS. Moreover, oocytes produced from neonatal ovaries by 2-step culture with FBS-containing medium during the whole period have a greater potential to develop to term than those produced by Eppig *et al.* 22,62,66 It is possible that various growth factors supplied by FBS are necessary in the culture medium. However, chemically defined medium is essential for increasing our understanding of the mechanisms of oogenesis.

In recent studies, PGCLCs have been differentiated from mouse embryonic stem cells and induced pluripotent stem (iPS) cells^32^ and successfully developed to fertile oocytes *in vitro*.^35^ Accordingly, the complete reconstitution of the process from non-germline cells to female germ cells can be accomplished *in vitro*. Since fertile oocytes can be produced from mitotically divided cells, such an *in vitro* system would expand the possibilities of the mass production of mammalian oocytes, sequential observations of oogenesis, and gene modifications during oogenesis using genome editing, RNA interference, or transfection technologies.

A culture system for PGCs has the potential to be applied to livestock and other mammals, but PGCs in these taxa are not well-characterized compared to those of mice. In pigs, the differentiation of PGCLCs to spermatogonial stem cell-like cells, but not spermatozoa, was observed after injection into busulfan-treated mouse testes.^101^ It may be possible to obtain functional oocytes in large animals from PGCs or PGCLCs; however, optimal culture conditions, including ICI addition and its concentration, should be examined in each species and at each age. Even in mice, there is variation in hormone levels and the timing of primordial follicle formation among strains.^77^ In bovines, estradiol has inhibitory effects on primordial follicle assembly,^105^ but promotes follicle formation in hamsters and baboons.^98^,^100^ Thus, although a prototype *in vitro* system to produce functional oocytes from PGCs and PGCLCs has been established in mice, further investigation is required for establish a system that is widely applicable across taxa.

The introduction of our *in vitro* system to human PGC culture is impractical. Human PGCs differentiate into oocytes by 2 months post-conception, and primordial follicle formation starts by 6 months post-conception^5^; therefore, there are no PGCs in adult ovaries. Recently, PGCLCs have been established from human iPS cells supplemented with BMP4, LIF, SCF, and EGF.^86^ However, differentiation of oocytes and spermatozoa from PGCLCs currently requires aggregation with somatic cells from embryonic gonads, at least in mice.^32^,^33^,^35^ If possible, it might take far longer to produce mature oocytes from human PGCs/PGCLCs. Even in follicle culture, it takes over 30 days to grow small antral follicles from secondary follicles *in vitro*,^96^ and there is no evidence for the development of human preantral follicles beyond Graafian follicles *in vitro*. At all steps, the culture of human PGCs/PGCLCs to produce mature oocytes raises ethical issues and safety concerns that have not been addressed.

For the last two decades, the existence of oogonial stem cells (OSCs) in adult ovaries has been a controversial topic. This idea stems from the discrepancy between the estimated number of oocytes in neonatal ovaries and the estimated number of ovulated oocytes and atretic follicles. Johnson *et al.* indicated that faster depletion of oocytes in the ovaries would be caused by a higher number of atretic follicles and ovulated oocytes if neo-oogenesis does not progress to adulthood.^40^ An increasing number of reports has supported the existence of OSCs in the adult ovaries of mice, rats, bovines, and humans.^20^,^30^,^40^,^46^,^74^ In these reports, OSCs are collected from adult ovaries by live-cell sorting using fluorescent- or magnetic-activated cell sorting (FACS or MACS) with germ cell or stem cell markers, such as MVH (known as Ddx4).^102^ Although the ratio of sorted OSCs after FACS or MACS was low in these studies, OSCs proliferated with the expression of both germ cell and stem cell markers during culture, and contributed to oocytes after grafting in ovaries * ex vivo*. However, the use of an MVH antibody in a live-cell sorting assay to detect antigens on the cell surface is questionable because MVH is a germline-specific RNA helicase and is believed to exist in the cytoplasm.^13^,^25^,^108^ Several attempts to resolve this issue with the MVH antibody approach have been reported using an SSEA-1 antibody or Ddx4-cre transgenic mice.^42^,^75^ However, it is still not clear whether OSCs exist. Our *in vitro* system could be used to evaluate whether OSCs exist to supply new oocytes to adult ovaries without transplantation of OSCs.

Thus, we demonstrated the fertility of mouse oocytes produced from PGCs *in vitro*. This new methodological approach has important implications for female germ cell preservation and studies of every process involved in mammalian oogenesis.
